# Supplementary material for: Supporting workflow reproducibility by linking bioinformatics tools across papers and executable code
Source: Bioinformatics. 2026 Jul 30;42(8):btag565. doi: 10.1093/bioinformatics/btag565 (PMC13489712; doi:10.1093/bioinformatics/btag565)
Supplement: Supplementary file 1 [file btag565_supplementary_data.pdf]

Supplementary Data For Paper: Supporting Workflow Reproducibility by Linking Bioinformatics Tools across Papers and Executable Code

Clémence Sebe, Olivier Ferret, Aurélie Névéol, Mahdi Esmailoghli,
Ulf Leser, Sarah Cohen-Boulakia

A Encoder-based approach for Named Entity Recognition

Figure 1 presents the BiLSTM-CRF approach.

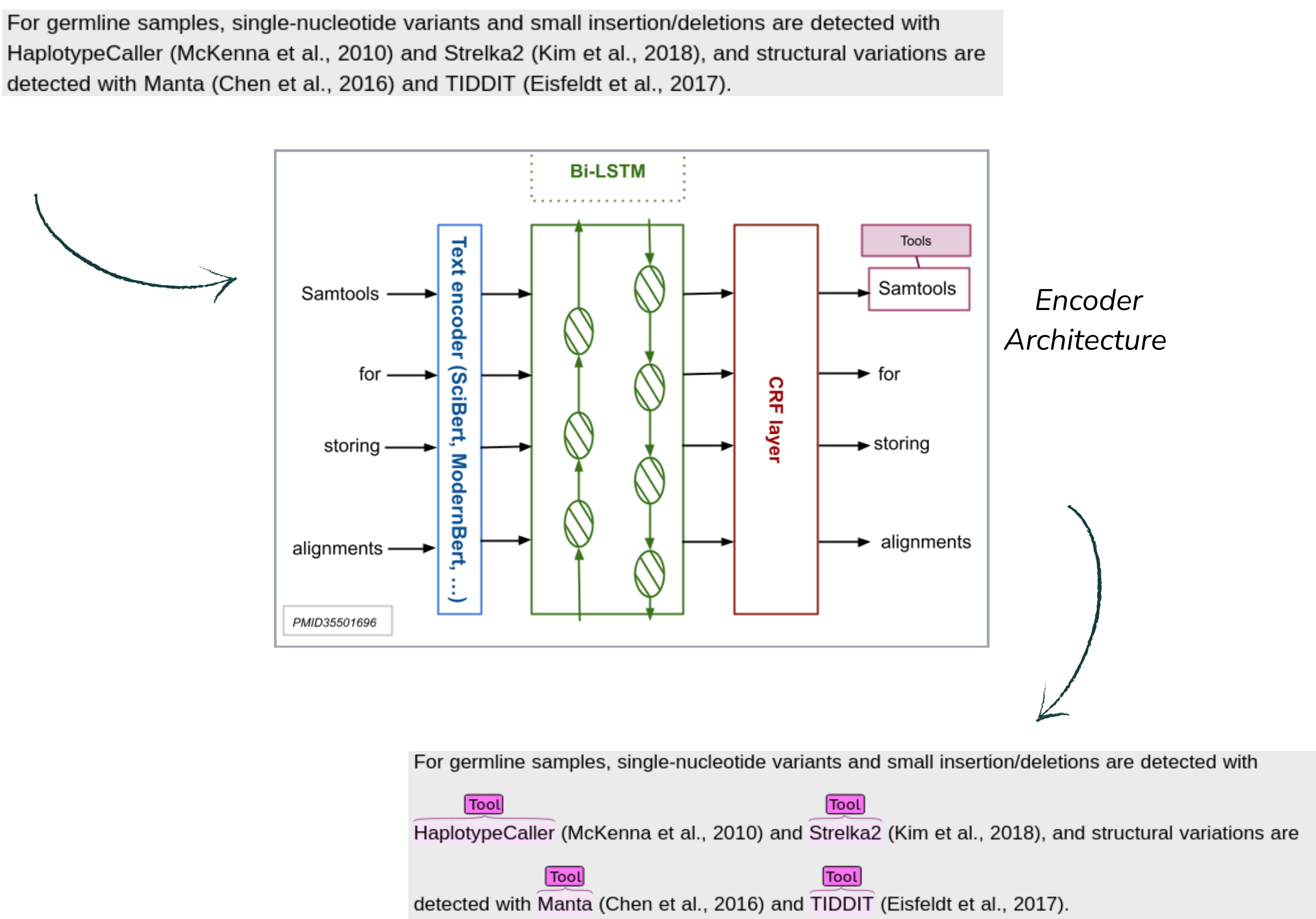

Figure 1: Encoder-based approach for NER.

B Named entity recognition: KB-based approach

Table 1: Performance of bioinformatics tool name extraction in terms of precision, recall, and F-measure (F1), using KBs.

Knowledge bases	CPL-Article			CPL-Code		
	Precision	Recall	F1	Precision	Recall	F1
Biocontainers	63.8	25.8	36.7	**67.6**	**36.6**	**47.5**
Bioconda	**34.0**	**70.0**	**45.7**	15.0	77.2	25.2
Biotools	20.0	60.0	30.0	10.5	56.9	17.7
Bioweb	26.2	51.2	34.7	24.5	64.0	35.4
Fusion	16.8	78.5	27.7	9.7	83.2	17.4

C Intermodal entity resolution

C.1 String-to-string comparison

Table 2: Performance using string-to-string comparison.

	Precision	Recall	F1
String-to-string	*74.6*	*75.7*	*75.1*
Levenshtein1	74.8	78.5	76.6
Levenshtein2	73.4	80.6	76.8
Levenshtein3	65.2	82.6	72.9
**Prefix/Suffix**	**73.9**	**82.6**	**78.0**

C.2 Using knowledge bases as a bridge

Table 3: Performance obtained using Bioconda-Bioweb-fusion and its fusion with string-to-string comparison methods.

	Precision	Recall	F1
**Bioconda-Bioweb-fusion-exact**	**81.2**	**82.1**	**81.6**
Bioconda-Bioweb-fusion-Lev1	79.5	80.5	80.0
Bioconda-Bioweb-fusion-Lev2	77.0	78.0	77.5
Bioconda-Bioweb-fusion-Prefixe/Suffixe	74.7	75.8	75.2

D Decoder-based approach

Table 4: Performance of bioinformatics tool name extraction in terms of precision, recall, and F-measure (F1) using the decoder-based approach.

Corpus	Model	Precision	Recall	F1
CPL-Article	Llama-3.2-3B-Instruct (Grattafiori et al., 2024)	61.1	73.8	66.8
	Llama-3.1-8B-Instruct (Grattafiori et al., 2024)	63.8	78.2	70.3
	Llama-3.1-70B-Instruct (Grattafiori et al., 2024)	75.0	52.4	61.7
	Llama-3.3-70B-Instruct (Grattafiori et al., 2024)	67.5	61.1	64.2
	Qwen3-4B-Instruct-2507 (Team, 2025)	64.8	43.9	52.3
	Qwen3-8B (Team, 2025)	65.6	73.7	69.4
	Qwen3-14B (Team, 2025)	63.1	57.7	60.3
CPL-Code	CodeLlama-13b-Instruct-hf (Rozière et al., 2024)	40.5	13.7	20.5
	CodeLlama-70b-hf (Rozière et al., 2024)	24.5	13.8	17.7
	CodeLlama-70b-Instruct-hf (Rozière et al., 2024)	55.2	11.5	19.0
	Llama-3.2-3B-Instruct (Grattafiori et al., 2024)	43.8	16.9	24.4
	Llama-3.1-8B-Instruct (Grattafiori et al., 2024)	39.5	17.3	24.1
	Llama-3.1-70B-Instruct (Grattafiori et al., 2024)	50.7	21.2	29.9
	Llama-3.3-70B-Instruct (Grattafiori et al., 2024)	40.5	15.9	22.9
	Qwen3-4B-Instruct-2507 (Team, 2025)	41.9	18.1	25.3
	Qwen3-8B (Team, 2025)	43.4	18.2	25.6
	Qwen3-14B (Team, 2025)	61.8	14.1	22.9
	Qwen2.5-Coder-14B-Instruct (Hui et al., 2024; Yang et al., 2024)	63.5	20.3	30.7
	Qwen3-Coder-30B-A3B-Instruct (Team, 2025)	36.7	26.3	30.7

E Environmental impact

The environmental impact of the methods was assessed using Global Warming Potential (GWP) expressed in gCO_2e using Green Algorithms[1] (Lannelongue et al., 2021). Each NER experiment was conducted on NVIDIA RTX A6000, Tesla V100 or NVIDIA H100 GPU. Our results show that encoder-based models exhibit a higher initial carbon footprint (about $80gCO_2e$ per run for a base model, about $100gCO_2e$ per run for a model with vocabulary injection) than decoder-based models (about 3 gCO_2e per run). However, this impact must be considered in relation to task performance and the models' life cycle. As seen in Tables 6-7 encoder based models yield higher F1 for both article (77 vs. 70) and code (90 vs. 30). In addition, encoder-based models were trained specifically for our experiments, so the impact of training was included in the GWP assessment, while we considered it negligible for decoder models. Even though the inference cost per sentence processed was lower than for decoder-based models, encoder models' training created an overhead that led to the higher overall carbon footprint. In contrast, decoder-based models are used in a few-shot setting, and since we used existing models in our experiments, we did not account for the overhead impact of training them. These results contrast with the findings of Naguib et al. (2024), who used a substantially larger corpus, in which the cumulated impact of inferences using decoder models exceeded the cost of encoder models' training by far.

References

A. Grattafiori, A. Dubey, A. Jauhri, A. Pandey, A. Kadian, A. Al-Dahle, A. Letman, A. Mathur, A. Schelten, A. Vaughan, A. Yang, A. Fan, and et al. The llama 3 herd of models. 2024. arXiv:2407.21783 [cs.AI].

B. Hui, J. Yang, Z. Cui, J. Yang, D. Liu, L. Zhang, T. Liu, J. Zhang, B. Yu, K. Dang, et al. Qwen2. 5-coder technical report. *arXiv preprint arXiv:2409.12186*, 2024.

L. Lannelongue, J. Grealey, and M. Inouye. Green algorithms: Quantifying the carbon footprint of computation. *Advanced Science*, 8(12):2100707, 2021. doi: 10.1002/advs.202100707.

M. Naguib, X. Tannier, and A. Névéol. Few-shot clinical entity recognition in English, French and Spanish: masked language models outperform generative model prompting. In *Findings of EMNLP*, 2024.

B. Rozière, J. Gehring, F. Gloeckle, S. Sootla, I. Gat, X. E. Tan, and et al. Code llama: Open foundation models for code. 2024. arXiv:2308.12950 [cs.CL].

Q. Team. Qwen3 technical report. 2025. arXiv:2505.09388 [cs.CL].

A. Yang, B. Yang, B. Hui, B. Zheng, B. Yu, C. Zhou, C. Li, C. Li, D. Liu, F. Huang, G. Dong, H. Wei, H. Lin, J. Tang, and et al. Qwen2 technical report. *arXiv preprint arXiv:2407.10671*, 2024.

[1] `https://calculator.green-algorithms.org/`
